# The atypical dual-specificity protein phosphatase (DUSP)/kinatase of *Leishmania infantum* modulates infectivity, oxidative stress response and antimonial resistance

**DOI:** 10.1371/journal.pntd.0014330

**Published:** 2026-05-26

**Authors:** Juliana Martins Ribeiro, Giovanna Ferreira da Fonseca, Milena Airi Kimura Anbo, Karla Ferreira Costa, Amanda Cristina Silva de Oliveira, Karine Ferreira Lopes, Mariza Gabriela Faleiro de Moura Lodi Cruz, Héllida Marina Costa-Silva, Ana Maria Murta Santi, Silvane Maria Fonseca Murta

**Affiliations:** 1 Fundação Oswaldo Cruz - Fiocruz, Instituto René Rachou, Belo Horizonte, Minas Gerais, Brazil; 2 Fundação Oswaldo Cruz - Fiocruz, Instituto Carlos Chagas, Curitiba, Paraná, Brazil; Centro de Pesquisa Gonçalo Moniz-FIOCRUZ/BA, BRAZIL

## Abstract

Dual-specificity protein phosphatases (DUSPs) are key modulators of mitogen-activated protein kinase (MAPK) signaling pathways that regulate cellular proliferation, differentiation, and stress adaptation. Although extensively characterized in higher eukaryotes, the functional roles of DUSPs in *Leishmania* remain poorly understood. In this study, we investigated the function of an atypical dual-specificity phosphatase/kinatase (LINF_340027100) in *Leishmania infantum* using CRISPR/Cas9-mediated genome editing. Repeated attempts to generate *DUSP*-null mutants were unsuccessful, and episomal complementation prior to chromosomal disruption did not permit deletion of the endogenous locus, suggesting that *DUSP* is essential for promastigote viability. To investigate DUSP function, we generated parasite lines with altered gene expression, including a heterozygous knockout (*Li* DUSP^+/−^) and an episomal overexpressor (*Li* WT + pIR1_SAT-DUSP). Modulation of *DUSP* expression resulted in pronounced alterations in parasite morphology, cell-cycle progression, differentiation, and intracellular proliferation in macrophages. The *Li* DUSP^+/−^ line exhibited a twofold reduction in transcript abundance and maintained normal growth kinetics, but displayed elongated morphology, increased flagellar length, and a reduced G0/G1 population with concomitant S-phase accumulation. In addition, *Li* DUSP^+/−^ parasites showed decreased tolerance to hydrogen peroxide, being 1.5- and 5.7-fold more sensitive in promastigote and intracellular amastigote forms, respectively. Conversely, the *DUSP*-overexpressing line exhibited a 2.3-fold increase in *DUSP* transcript levels, accompanied by modest impairment of promastigote proliferation, rounded morphology, altered cell-cycle distribution, and increased resistance to trivalent antimony, with 2.6- and 3.1-fold higher resistance observed in promastigote and intracellular amastigote forms, respectively. Both mutant lines showed reduced intracellular proliferation at 72 h post-infection. Transcript level analysis revealed altered expression of *MAPK1*, *MAPK3*, and *MAPK10* in both promastigote and axenic amastigote-like forms, supporting a functional link between DUSP activity and MAPK signaling homeostasis. Together, these findings identify DUSP as a key regulator of parasite biology that integrates MAPK signaling with oxidative stress response, differentiation, and antimony susceptibility in *L. infantum*. This study provides the first functional characterization of this atypical DUSP/kinatase in *Leishmania* and highlights its potential as a target for therapeutic intervention.

## Author summary

Leishmaniasis is a neglected tropical disease that affects millions of people worldwide and can be fatal if left untreated. Current treatments are limited, often toxic, and increasingly compromised by drug resistance. Understanding how *Leishmania* parasites regulate essential cellular processes is crucial for identifying new therapeutic targets. Dual-specificity phosphatases (DUSPs) are enzymes that control protein phosphorylation and play central roles in cell signaling, growth, and stress responses. However, their function in *Leishmania* remains largely unknown. In this study, we investigated the role of an atypical DUSP/Kinatase in *Leishmania infantum* by creating mutant parasites with reduced or increased gene expression. Complete deletion of the gene was not possible, suggesting that this *DUSP* is essential for parasite survival. Reduced *DUSP* expression was associated with elongated morphology, disrupted cell-cycle progression, and increased sensitivity to oxidative stress, whereas *DUSP* overexpression led to a rounded morphology and enhanced resistance to antimony, a key antileishmanial drug. These results reveal that *DUSP* is a critical regulator of signaling and redox balance in *L. infantum*, influencing parasite morphology, cell-cycle progression, and drug response. Our findings highlight DUSP as a potential target for developing new therapies against leishmaniasis.

## Introduction

*Leishmania* spp. are protozoan parasites that cause leishmaniasis, a group of neglected tropical diseases affecting millions of people worldwide [1]. *Leishmania infantum*, the etiological agent of visceral leishmaniasis across the Mediterranean Basin, the Middle East, Central Asia, South America, and Central America, is responsible for the most severe form of the disease, which can be fatal without timely therapeutic intervention [2]. Currently, no vaccines for human use are available, and the absence of effective vector control strategies makes chemotherapy the primary approach for managing all clinical manifestations of leishmaniasis. Existing therapeutic agents — including pentavalent antimonials, amphotericin B formulations, and miltefosine — remain indispensable; however, their efficacy is increasingly challenged by emerging reports of parasite resistance, and the adverse effects associated with current regimens [3]. These limitations highlight the urgent need to identify novel molecular targets to support the development of safer and more effective therapeutic alternatives.

*Leishmania* parasites alternate between two main developmental forms: the motile promastigote stage within the sandfly vector and the intracellular amastigote stage that resides in mammalian macrophages [2,4]. Successful adaptation of *Leishmania* to these contrasting environments relies on the dynamic regulation of signaling networks that orchestrate proliferation, differentiation, and responses to environmental stress. Among these regulatory systems, the mitogen-activated protein kinase (MAPK) cascade plays a pivotal role in coordinating parasite survival, virulence, and drug resistance [5]. MAPK activity is precisely controlled through cycles of phosphorylation and dephosphorylation. DUSPs of *Leishmania* comprise a diverse superfamily that include lipid phosphatases [6,7], as well as protein phosphatases capable of dephosphorylating both serine/threonine and tyrosine residues (including eukaryotic-like DUSPs, atypical DUSPs, and uncharacterized kinetoplastid-specific DUSPs) [8,9,10]. They share a conserved catalytic signature motif, HCX₅R, in which the nucleophilic cysteine and adjacent arginine residues are essential for substrate recognition and catalysis [9,10]. By fine-tuning the intensity and duration of MAPK signaling [8,10], DUSPs are also key enzymes that play a central role in wide range of cellular processes, including cell cycle progression, apoptosis, and adaptation to oxidative stress [11–13].

Elucidating how DUSPs modulate MAPK pathways in *Leishmania* may uncover key mechanisms underlying parasite survival and stress adaptation, as well as potential targets for therapeutic intervention. Notably, transcriptomic analyses comparing wild-type and trivalent antimony (Sb^III^)-resistant *L. infantum* lines revealed significant differential expression of genes associated with the resistance phenotype, including an 8.9-fold upregulation of *DUSP* (LINF_340027100) transcripts in the Sb^III^-resistant line [14]. Furthermore, this DUSP is also secreted within *Leishmania* exosomes and may directly modulate host cell phosphorylation cascades during infection [15,16].

To further investigate the role of the atypical DUSP/Kinatase (LINF_340027100), we generated *L. infantum* mutant lines with altered *DUSP* expression levels. These lines were subsequently characterized with respect to growth, morphology, infectivity, and drug susceptibility. Hereafter, the gene, its transcript, and the corresponding protein product are collectively referred to as “DUSP” for simplicity.

## Methodology

### Parasites cultures conditions

Promastigote forms of *Leishmania* (*Leishmania*) *infantum* (MHOM/BR/1974/PP75) expressing red fluorescence (tdTomato) [17] and/or constitutively expressing Cas9 (plasmid pTB007; [18]) were cultured at 26°C in M199 medium (Gibco – Thermo Fischer Scientific, Grand Island, NY, USA) supplemented with the following components: 40 mM HEPES (pH 7.4), 5 μg/mL hemin, 2 μg/mL biopterin, 1 μg/mL biotin, 2 mM L-glutamine, 500 U/mL penicillin, 50 μg/mL streptomycin, and 10% heat-inactivated fetal bovine serum. Cultures were maintained by biweekly passages, inoculating 1 × 10^6^ parasites per 5 mL of medium.

### Generation of *DUSP*-overexpressing *L. infantum* mutants

*DUSP*-overexpressing *L. infantum* promastigotes expressing tdTomato were generated using the pIR1_SAT expression vector. Briefly, the *DUSP* coding sequence (TritrypDB: LINF_340027100) was amplified by PCR using Phusion High-Fidelity DNA Polymerase (Thermo Fisher Scientific, Grand Island, NY, USA) and assembled into the SwaI-linearized pIR1_SAT vector using Gibson Assembly (New England Biolabs, Ipswich, MA, USA). Primers (S1 Table) were designed to generate 25–30 bp overlaps between the insert and the vector backbone using the NEBBuilder Assembly Tool. The assembly reaction was transformed into chemically competent *Escherichia coli* TOP10F’ cells. Positive clones were screened by colony PCR and confirmed by Sanger sequencing. Parasites were transfected with circular plasmid form (New England Biolabs, Ipswich, MA, USA) and selected with 50 μg/mL nourseothricin sulfate (Sigma-Aldrich - Merck, St. Louis, MO, USA) to establish stable overexpressing lines. Transfections were performed as previously described by [19]. All primers used for Gibson Assembly construction are listed in S1 Table.

### Attempt to generate *DUSP L. infantum* knockout

The initial attempt to generate *DUSP* (Tritryp DB: LINF_340027100) knockout *L. infantum* parasites was carried out using the CRISPR/Cas9 system [18]. Promastigotes expressing tdTomato, Cas9, and T7 RNA polymerase were co-transfected with PCR-amplified sgRNA and donor DNA fragments, which were selected using the LeishGEdit tool. Donor DNAs were amplified by PCR with specific primer pairs using plasmids pTNEO_v1 and pTPURO_v1 as templates, which confer resistance to neomycin (NEO) (Geneticin, G418 Sulfate, Gibco – Thermo Fischer Scientific, Grand Island, NY, USA)) and puromycin (PURO) (Sigma-Aldrich - Merck, St. Louis, MO, USA), respectively. For transfections, promastigotes were electroporated with equimolar amounts of sgRNAs targeting the 5′ and 3′ untranslated regions (UTRs) along with the corresponding donor DNAs. Parasites were subsequently selected using combinations of 40 μg/mL NEO with 10, 15, or 20 μg/mL PURO. Heterozygous knockout line (*Li* DUSP^+/-^) was obtained by transfecting parasites with sgRNAs targeting the 5′ UTR and 3′ UTR and the NEO donor DNA alone, followed by selection with 40 μg/mL NEO.

In a subsequent attempt to generate endogenous *DUSP*-deficient mutants, the tdTomato- and Cas9-expressing *L. infantum* line was first transfected with the pIR1_SAT-DUSP vector, which carries nourseothricin resistance as a selectable marker, as described above. The pIR1_SAT-*DUSP* vector was used in its circular form without prior digestion. Following selection with 50 μg/mL nourseothricin sulfate, parasites were transfected with donor DNA fragments and sgRNA templates designed to disrupt the chromosomal *DUSP* locus, aiming to obtain either complete or single-knockout lines. Donor DNAs were amplified by PCR using plasmids pTNeo v1 and pTPuro v1 as templates, conferring resistance to neomycin (NEO) and puromycin (PURO), respectively. Parasites were selected using either 15 μg/mL PURO plus 40 μg/mL NEO, or 20 μg/mL PURO plus 60 μg/mL NEO for *DUSP* null mutant lines selection. All transfections were performed as previously described [19]. All primers used for plasmid and DNA fragment construction are listed in S1 Table.

### Quantification of transcript levels

To assess transcript levels of the target gene, quantitative reverse-transcription PCR (RT-qPCR) analysis was performed using cDNA from mutants and *L. infantum* controls parasites. Promastigotes (approximately 1 × 10⁸ cells) were resuspended in 1 mL of TRIzol Reagent (Invitrogen - Thermo Fisher Scientific, Waltham, MA, USA), and total RNA was extracted using the chloroform phase-separation method. The RNA samples were treated with the Turbo DNA-free Kit (Invitrogen - Thermo Fisher Scientific, Waltham, MA, USA) to remove residual genomic DNA, and complementary DNA (cDNA) was synthesized with SuperScript II Reverse Transcriptase (Invitrogen - Thermo Fisher Scientific, Waltham, MA, USA) according to the manufacturer’s instructions

cDNA samples were diluted to a final concentration of 100 ng/μL and used for amplification reactions with Power SYBR Green Master Mix (Applied Biosystems - Thermo Fisher Scientific, Waltham, MA, USA). Specific primers used for *DUSP* quantification are listed in S1 Table. Amplifications were performed in triplicate in a 96-well plate using the QuantStudio 12 Flex Real-Time PCR System (Thermo Fisher Scientific, Waltham, MA, USA) under the following cycling conditions: 50°C for 2 min and 95°C for 10 min, followed by 40 cycles of 95°C for 15 s and 60°C for 1 min, and a final dissociation stage at 95°C for 15 s, 60°C for 15 s, and 95°C for 15 s.

The constitutive DNA polymerase I gene (Tritryp DB: LINF_160021500) was used as an endogenous reference for normalization. Relative quantification of mRNA expression was calculated using the comparative Ct method (2^⁻ΔΔCt^) [20]. Three independent biological replicates were performed for each parasite line, with each replicate analyzed in technical quadruplicate.

### Growth curve of promastigotes forms

*L. infantum* promastigote forms (1 × 10^5^ parasites/mL) were cultured in M199 medium (Gibco – Thermo Fischer Scientific, Grand Island, NY, USA) at 26°C, and parasite density was monitored daily using a Z1 Coulter Particle Counter (Beckman Coulter, Brea, CA, USA). All assays were carried out in triplicate across three independent biological experiments.

### Ficoll enrichment of metacyclic parasites

Promastigote cultures of *Li* Cas9, *Li* DUSP^+/−^, *Li* WT, and *Li* WT + pIR1_SAT-DUSP (1 × 10⁵ parasites/mL) were maintained at 26 °C for five days. Parasites were subsequently collected, and metacyclic forms were isolated by Ficoll gradient centrifugation according to [21], with minor modifications. Briefly, Ficoll 400 (Sigma-Aldrich - Merck, St. Louis, MO, USA) was used to prepare a 20% stock solution in PBS and diluted for preparation of 10% Ficoll solution. Two ml of 20% Ficoll were overlaid by 2 ml of 10% Ficoll and 2 ml of parasite suspension were layered on top of the Ficoll cushion. Tubes were centrifuged at 2,500 rpm for 15 min at room temperature without brake. The metacyclic-enriched fractions were recovered at the interface between the 20% and 10% Ficoll layers. Parasites were washed with PBS, and cell numbers were determined using a Neubauer hemocytometer. Three independent experiments were performed in duplicate.

### Morphological and morphometric analysis of *L. infantum* promastigotes

Promastigotes from control and mutant *L. infantum* lines were collected at 48 and 96 hours after the initial inoculation (1 × 10⁵ parasites/mL) and washed twice with phosphate-buffered saline (PBS, pH 7.4). Parasite suspensions were then applied to glass slides, allowed air dry, and fixed with methanol for 5 minutes. Fixed slides were stained with 10% Giemsa solution (Sigma-Aldrich - Merck, St. Louis, MO, USA) for 15–20 minutes, rinsed gently with distilled water, and air-dried. Parasite morphology was evaluated under a light microscope (Axio Observer.A1, Zeiss, Germany) at 63 × magnification using oil immersion, and images were captured using a digital camera attached to the microscope.

Morphometric analyses of promastigotes from control and mutant *L. infantum* lines were performed using digital images obtained by light microscopy. Flagellar and cell body lengths were measured using ImageJ software (National Institutes of Health, USA). For each experimental group, 50 individual parasites were randomly selected and analyzed. Flagellar length was measured from the flagellar pocket to the distal tip of the flagellum, and cell body length was measured along the longitudinal axis of the parasite. Measurements were obtained from two independent experiments performed in duplicate.

### Cell cycle analysis

Promastigotes (1 x 10^6^ parasites) of *L. infantum* (control and mutants) were harvested after 48 and 96 hours of growth (initial inoculation of 1 x 10^5^ parasites/mL), washed twice with phosphate-buffered saline (PBS, pH 7.4), and fixed in 70% ethanol at 4°C overnight. Fixed cells were washed with PBS and incubated for 30 minutes at room temperature in the dark with a staining solution containing propidium iodide (PI, 50 μg/mL; Sigma-Aldrich - Merck, St. Louis, MO, USA) and RNase A (100 μg/mL; Sigma-Aldrich, St. Louis, MO, USA) to simultaneously stain DNA and remove RNA [22]. Cell cycle distribution was analyzed using flow BD FACSCalibur flow cytometer (BD Biosciences, San Jose, CA, USA), acquiring 30,000 events per sample. Data were processed using FlowJo software (v10), and the percentage of cells in sub G0/G1, G0/G1, S, and G2/M phases was determined. Only singlet events were considered for the analysis of DNA content and cell cycle distribution. Experiments were performed in three independent biological replicates.

### Infection of THP-1 cell-derived macrophages

THP-1 cells (a human monocyte lineage) were cultured in RPMI-1640 (Sigma-Aldrich - Merck, St. Louis, MO, USA) medium supplemented with 2 mM glutamine, 100 U/mL penicillin, 100 μg/mL streptomycin, and 10% fetal bovine serum, and maintained at 37°C in a 5% CO₂ atmosphere. To induce differentiation into macrophages, cells were treated with 20 ng/mL phorbol myristate acetate (PMA; Sigma-Aldrich - Merck, St. Louis, MO, USA) and incubated in black 96-well plates under the same conditions. After 72 hours, differentiated macrophages were infected with *Leishmania* promastigotes from the second day of the stationary phase at a ratio of 20 parasites per macrophage for 4 hours. Non-internalized parasites were removed by washing, and the infected macrophages were then maintained in RPMI-1640 medium for 72 hours. The intracellular amastigote development was assessed at 4- and 72-hours post-infection by measuring fluorescence intensity using a microplate reader (Variokan Lux, Thermo Fisher Scientific, Waltham, MA, USA) (excitation at 554 nm and emission at 581 nm) [17].

### Effective concentration (EC_50_) assays in promastigotes

Parasite susceptibility to different compounds was assessed using *L. infantum* promastigotes from both control and mutant lines (2 × 10⁶ parasites/mL) cultured in 1 mL of M199 medium containing increasing concentrations of each compound: 25–350 μM potassium antimony tartrate (Sigma-Aldrich, St. Louis, MO, USA), 0.05-0.4 μM amphotericin B (Sigma-Aldrich, St. Louis, MO, USA), 5–40 μM miltefosine (Cayman Chemical, Ann Arbor, MI USA), and 30–210 μM hydrogen peroxide (Sigma-Aldrich - Merck, St. Louis, MO, USA). After 48 hours of incubation at 26°C, parasite viability was evaluated by measuring the fluorescence intensity of tdTomato-expressing parasites using a microplate fluorescence reader (Variokan Lux, Thermo Fisher Scientific, Waltham, MA, USA) with excitation and emission wavelengths of 554 nm and 581 nm, respectively [17]. The half-maximal effective concentration (EC₅₀) values were calculated by non-linear regression using GraphPad Prism 8.2.0 from data obtained in three independent biological replicates, each performed in triplicate.

### Effective concentration (EC_50_) assays in intracellular amastigotes

Following infection as described above, THP-1 cell–derived macrophages were incubated in RPMI-1640 medium containing increasing concentrations of trivalent antimony (2.3 – 300 µM) or hydrogen peroxide (1.2 – 600 µM) for 72 hours. After treatment, parasite viability was assessed by measuring the decrease in fluorescence intensity of tdTomato-expressing parasites using a fluorescence microplate reader (Variokan Lux, Thermo Fisher Scientific, Waltham, MA, USA) with excitation and emission wavelengths of 554 nm and 581 nm, respectively [17]. The half-maximal effective concentration (EC₅₀) values were calculated by non-linear regression using GraphPad Prism 8.2.0 from data obtained in three independent biological experiments, each performed in triplicate.

### Axenic amastigote-like culture for quantification of gene transcript levels

*L. infantum* promastigotes were differentiated into axenic amastigote-like forms following an established protocol [23,24] with minor modifications. Briefly, promastigotes in the stationary growth phase were harvested, washed twice with PBS, and resuspended in RPMI medium (Sigma-Aldrich - Merck, St. Louis, MO, USA) supplemented with 20% fetal bovine serum, 100 U/mL penicillin, and 100 μg/mL streptomycin. The pH was adjusted to 5.5 to mimic the phagolysosomal environment. Parasites were incubated at 37°C in a 5% CO₂ atmosphere. Axenic amastigote-like morphology and viability were monitored daily by light microscopy. Parasites were allowed to differentiate for 3–5 days, and the resulting axenic amastigote-like forms were used for subsequent analyses.

### Transcriptional analysis of MAPKs in promastigotes and axenic amastigote-like forms

The transcriptional levels of *MAPK1* (Tritryp DB: LINF_360076200), *MAPK3* (Tritryp DB: LINF_100011600), and *MAPK10* (Tritryp DB: LINF_100007100) were assessed in *L. infantum* promastigote and axenic amastigote-like forms of mutants using RT-qPCR. cDNA was prepared as described above, and RT-qPCR reactions were carried out under the same conditions, with the only difference being the use of gene-specific primers for each MAPK (S1 Table). Relative transcript levels were calculated using the comparative Ct (2^⁻ΔΔCt^) method, with the constitutive DNA polymerase I gene serving as an endogenous reference. All reactions were performed in triplicate across three independent biological replicates.

### Statistical analysis

Data were analyzed using GraphPad Prism version 8.2.0 (San Diego, CA, USA). Comparisons between *Li* Cas9 and *Li* DUSP^+/-^ or *Li* WT and *Li* WT + pIR1_SAT-DUSP parasites were performed using ordinary one-way or two-way ANOVA followed by Bonferroni’s post hoc test. Analysis between two groups was performed using t-test. Differences were considered statistically significant at p < 0.05.

## Results

### Genomic Localization and Predicted Structure of the DUSP/Kinatase LINF_340027100

A search using the TriTrypDB resource [25] for DUSP LINF_340027100 reveals that it corresponds to LmjF.34.2190, a protein identified as an atypical DUSP “Kinatase” [9]. A protein-level comparison of LINF_340027100 from *L. infantum* and LmjF.34.2190 from *L. major* is presented in S1 Fig, which shows a CLUSTAL O (1.2.3) multiple sequence alignment. The genome of the *L. infantum* JPCM5 strain encodes a single copy of the atypical DUSP/Kinatase gene. This gene spans 4,149 base pairs and encodes a protein of 1,382 amino acids.

Analysis of the LINF_340027100 protein sequence using PFAM data retrieved from TriTrypDB (release 68, May 2024) revealed a multi-domain architecture characteristic of atypical kinatases (S2A Fig). The protein contains a single Protein Kinase domain (PF00069; Pkinase), a Dual-Specificity Phosphatase catalytic domain (PF00782; DSPc), and a Leucine-Rich Repeat domain (PF13855; LRR_8). Consistent with this annotation, the protein is assigned to the KEGG orthologous group K14165, which corresponds to atypical DUSPs with predicted phosphatase activity against phosphotyrosine, phosphoserine, and phosphothreonine substrates (EC 3.1.3.16; EC 3.1.3.48).

Structural predictions derived from the AlphaFold database (entry A4I9Z1) reveal a multi-domain architecture comprising seven distinct structural regions, with large portions of the model displaying high or very high confidence scores (pLDDT) (S2B and S2C Fig).

### Modulation of DUSP/Kinatase LINF_340027100 expression in *Leishmania infantum*: Generation of loss- and gain-of-function lines

An initial attempt to generate *DUSP* knockout *L. infantum* lines using the CRISPR/Cas9 system was unsuccessful, as no viable parasites were recovered after antibiotic selection, suggesting that *DUSP* is essential for promastigote survival. A second strategy was then employed, in which *DUSP* was first expressed episomally, followed by CRISPR/Cas9-mediated disruption of the chromosomal copy. Correct integration of the NEO and PURO selection cassettes was confirmed by PCR using primer pairs targeting the 5′ UTR region adjacent to the donor DNA and the coding sequences of NEO or PURO (S3A and S3B Fig). However, PCR analysis revealed that the endogenous *DUSP* locus was still present in these transfectants (S3C Fig). This outcome suggests the retention of at least one copy of *DUSP*, likely through aneuploidy or gene amplification, despite successful integration of both selectable markers. Collectively, these findings suggest that *DUSP* is indispensable for the viability of *L. infantum* promastigotes.

**Fig 1:**
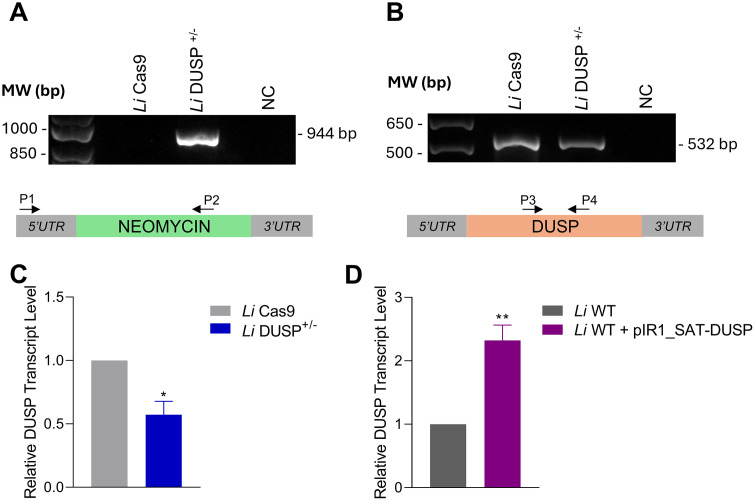
Generation of *L. infantum* Lines with altered DUSP/Kinatase LINF_340027100 expression. **(A)** The correct integration of the resistance marker neomycin in *Li* DUSP^+/-^ parasites (944 bp) was evaluated by PCR by annealing a primer in a 5′UTR region adjacent to the cassette (primer P1) and another primer annealed within resistance marker sequence (primer P2). **(B)** Fragment *DUSP*-coding sequence in *Li* DUSP^+/-^ parasites (532 bp) was amplified using PCR with primers P3 and P4. **(C)** Relative *DUSP* transcript level in *Li* DUSP^+/-^ parasites. **(D)** Relative *DUSP* transcript level in *Li* WT + pIR1_SAT-DUSP parasites. Relative *DUSP* transcript levels were determined by RT-qPCR. Relative quantification was performed using the 2^⁻ΔΔCt^ method, with the DNA polymerase gene used as an internal reference for normalization. T test was used to compare control parasite *Li* Cas9 and *Li* DUSP^+/-^ or *Li* WT and *Li* WT + pIR1_SAT-DUSP parasites. Data represents the mean ± standard deviation of three independent experiments performed in triplicates. * Represents significant differences in relation to the control parasite (* *p* < 0.05 and ** *p* < 0.01). MW: molecular weight; NC: negative control; bp: base pair.

In contrast, viable parasites were successfully obtained by deleting a single *DUSP* allele, resulting in the heterozygous knockout mutant *L. infantum* line *Li* DUSP^+/-^. This line was obtained by transfecting *L. infantum* Cas9-expressing parasites with sgRNAs targeting the 5′ and 3′ untranslated regions (UTRs) of *DUSP*, together with a donor cassette containing the neomycin resistance (NEO) gene for selection. Homologous recombination following sgRNA-directed cleavage at the target locus replaced one *DUSP* allele with the NEO cassette. Correct integration of the NEO cassette into the *L. infantum* genome was confirmed by PCR using primer P1, which anneals to the 5′ UTR of *DUSP*, and primer P2, specific to the NEO sequence (Fig 1A). Additional PCR using *DUSP*-specific primers confirmed the presence of a single intact allele in *Li* DUSP^+/-^parasites, consistent with the replacement of only one *DUSP* copy (Fig 1B). Quantitative analysis of transcript expression level of the *DUSP* in the mutant *Li* DUSP^+/-^ line is shown in Fig 1C. Deletion of one *DUSP* allele resulted in a 2.1-fold decrease in transcript expression levels.

Episomal overexpression of DUSP yielded viable *L. infantum lines*, designated *Li* WT + pIR1_SAT-DUSP, which exhibited a 2.3-fold upregulation of *DUSP* transcript levels (Fig 1D).

### DUSP/Kinatase LINF_340027100 modulation alters morphology, cell cycle, and metacyclogenesis in *L. infantum* promastigotes

**Fig 2:**
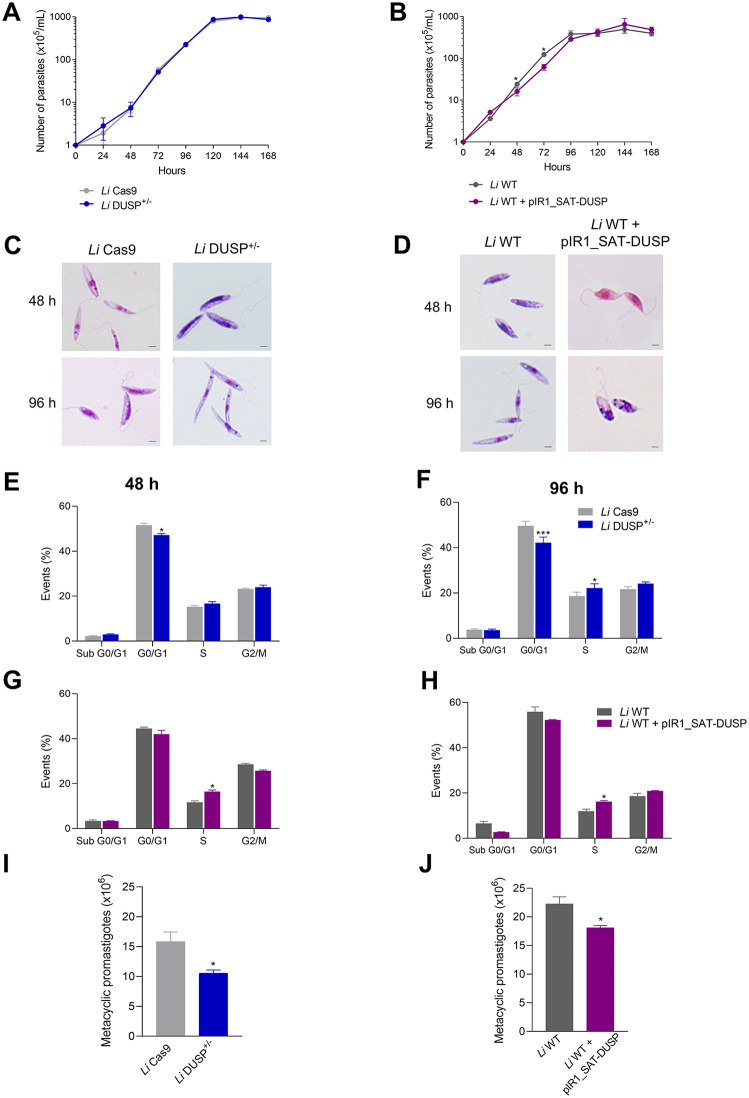
Growth curve, morphological and cell cycle analysis of promastigote forms of DUSP/Kinatase LINF_340027100 mutant parasites. An initial inoculum of 1 x 10^5^ parasites per mL was prepared for the *Li* Cas9, *Li* DUSP^+/-^, *Li* WT and *Li* WT + pIR1_SAT-DUSP parasites, which were counted every 24 h using the Z1 Coulter Counter. **(A)** Growth curve of *Li* Cas9 and *Li* DUSP^+/-^. **(B)** Growth curve of *Li* WT and *Li* WT + pIR1_SAT-DUSP. Morphological analysis of **(C)** *DUSP* heterozygous knockout and **(D)** overexpressing parasites after 48 hours and 96 hours of growth stained with Giemsa. **(E)** and **(F)** cell cycle analysis of *DUSP* heterozygous knockout at 48 hours and 96 hours, respectively. **(G)** and **(H)** cell cycle analysis of *Li* WT and *Li* WT + pIR1_SAT-DUSP parasites at 48 hours and 96 hours, respectively. The parasites were stained with propidium iodide and evaluated by flow cytometry. Quantification of metacyclic forms in *DUSP* heterozygous knockout **(I)** and *DUSP*-overexpressing parasites **(J)**. Metacyclic promastigotes were enriched using Ficoll gradient centrifugation. Data represents the mean ± standard deviation of three independent experiments performed in triplicates. Two-way ANOVA with Bonferroni’s post hoc test or T test was used to compare *Li* Cas9 and *Li* DUSP^+/-^, and *Li* WT and *Li* WT + pIR1_SAT-DUSP parasites at each time point. * Represents significant differences in relation to the control parasite (* *p* < 0.05 and *** *p* < 0.001). Image bars: 2 µm.

The growth of *Li* Cas9 and *Li* DUSP^+/-^ *L. infantum* mutants, as well as *Li* WT and *Li* WT + pIR1_SAT-DUSP promastigotes, was assessed by counting parasite numbers at 24-hour intervals. As shown in Fig 2A, *Li* DUSP^+/−^ parasites exhibited growth kinetics comparable to those of the *Li* Cas9 control line, indicating that loss of a single *DUSP* allele does not significantly impair parasite proliferation. In contrast, *DUSP*-overexpressing parasites showed a modest reduction in growth relative to *Li* WT controls, with statistically significant differences observed at two time points along the growth curve (Fig 2B). At 48 h, *Li* WT parasites reached an average density of 24.1 × 10⁵ parasites/mL, whereas *DUSP*-overexpressing parasites reached 16.2 × 10⁵ parasites/mL, corresponding to an approximate 32.7% reduction. A more pronounced difference was observed at 72 h, when *DUSP*-overexpressing parasites (~62.2 × 10⁵ parasites/mL) exhibited approximately 49.5% lower density than *Li* WT controls (~123.1 × 10⁵ parasites/mL). The complete dataset is presented in S2 Table.

Morphological analysis of promastigotes at 48 and 96 hours in culture, after Giemsa staining, revealed distinct phenotypic alterations associated with *DUSP* modulation. The heterozygous knockout line *Li* DUSP^+/−^ displayed an elongated and slender morphology at both points compared with the *Li* Cas9 control (Fig 2C). An increase in flagellar length was also observed at 48 hours. Quantitative morphometric analysis confirmed these observations. At 48 hours, *Li* DUSP^+/−^ parasites exhibited significantly longer flagella (15.9 ± 1.8 µm) than *Li* Cas9 parasites (8.2 ± 0.2 µm), as well as increased cell body length (14.9 ± 1.4 µm *vs* 8.6 ± 0.3 µm, respectively). Similar differences were observed at 96 hours, when *Li* DUSP^+/−^ parasites showed significantly greater cell body length than controls (13.9 ± 0.2 µm *vs* 11.8 ± 0.1 µm). No statistically significant difference in flagellar length was detected at 96 hours (9.0 ± 0.3 µm for *Li* DUSP^+/−^ *vs* 10.4 ± 0.5 µm for *Li* Cas9).

In contrast, *DUSP*-overexpressing parasites displayed a rounded cell body and reduced overall size, with significantly shorter flagella at both time points compared with *Li* WT controls (Fig 2D). Morphometric analysis confirmed this phenotype. At 48 hours, *DUSP*-overexpressing parasites exhibited significantly shorter flagella (6.4 ± 0.4 µm) than *Li* WT parasites (9.6 ± 0.2 µm), along with reduced cell body length (7.2 ± 0.6 µm vs 10.6 ± 0.5 µm). A similar pattern was observed at 96 hours, when overexpressing parasites showed significantly shorter flagella (8.2 ± 0.6 µm *vs* 12.7 ± 0.6 µm) and reduced cell body length (6.5 ± 0.4 µm *vs* 9.2 ± 0.4 µm) compared with *Li* WT controls. Together, these findings indicate that *DUSP* expression levels play a key role in maintaining promastigote morphology and flagellar architecture, likely through effects on cytoskeletal organization and dynamics.

Cell-cycle distribution of *L. infantum* promastigote lines was analyzed after 48 and 96 hours of cultivation in medium using propidium iodide staining. The heterozygous knockout *Li* DUSP^+/−^ line exhibited a reduced proportion of cells in the G0/G1 phase compared with *Li* Cas9 controls at 48 h (Fig 2E). This reduction persisted at 96 h and was accompanied by a corresponding accumulation of cells in S phase (Fig 2F). *DUSP*-overexpressing parasites also displayed an increased proportion of cells in S phase at both time points (48 and 96 h) compared with *Li* WT controls (Fig 2G-2H). Representative histograms are shown in S4 Fig. Collectively, these findings indicate that precise regulation of *DUSP* expression is required to maintain normal promastigote proliferation and proper cell-cycle progression.

Additionally, we evaluated whether modulation of *DUSP* expression affects metacyclogenesis. Metacyclic forms were enriched and quantified by Ficoll gradient centrifugation. Our results revealed a significant 33.5% reduction in the number of metacyclic forms in *Li* DUSP^+/−^ cultures compared with the *Li* Cas9 control (10.54 ± 0.96 × 10⁶ vs 15.85 ± 2.77 × 10⁶ parasites, respectively) (Fig 2I). Similarly, *DUSP*-overexpressing parasites exhibited a significant 18.7% reduction in metacyclic forms relative to *Li* WT controls (18.11 ± 0.69 × 10⁶ vs 22.27 ± 2.14 × 10⁶ parasites, respectively) (Fig 2J). These results indicate that both decreased and increased *DUSP* expression impair metacyclogenesis.

### Effect of *DUSP/Kinatase LINF_340027100* modulation on *L. infantum* infectivity in macrophages

**Fig 3:**
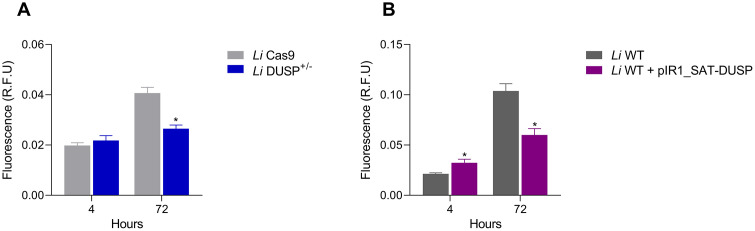
Analysis of the infectivity of DUSP/Kinatase LINF_340027100 heterozygous knockout and overexpressing parasites in THP-1 derived-macrophages. The fluorescence emission (R.F.U) of parasites **(A)** *Li* Cas9 and *Li* DUSP^+/-^ and **(B)** *Li* WT and *Li* WT + pIR1_SAT-DUSP after 4 and 72 hours of infection using THP-1 derived-macrophages. Two-way ANOVA with Bonferroni’s post hoc test was used to compare *Li* Cas9 and *Li* DUSP^+/-^, and *Li* WT and *Li* WT + pIR1_SAT-DUSP parasites at each time point. Data represents the mean ± standard deviation of three independent experiments performed in triplicates. * Represents significant differences in relation to the control (* *p* < 0.05).

The effect of *DUSP* modulation on parasite infectivity was assessed using THP-1–derived macrophages infected with *L. infantum* promastigotes, with intracellular parasite burden quantified by fluorescence intensity. After 4 hours of infection, *Li* DUSP^+/-^ parasites exhibited an infection profile comparable to that of the *Li* Cas9 control (Fig 3A). However, at 72 hours post-infection, a significant decrease in fluorescence was observed, indicating reduced intracellular replication of the *DUSP* heterozygous knockout mutant *Li* DUSP^+/-^ parasites (Fig 3A). In contrast, *DUSP*-overexpressing parasites displayed higher fluorescence at 4 hours post-infection (Fig 3B), suggesting enhanced macrophage entry, but markedly lower fluorescence after 72 hours, consistent with impaired intracellular replication relative to *Li* WT controls (Fig 3B). Collectively, these results suggest that both downregulation and overexpression of *DUSP* disturb the balance required for optimal *L. infantum* entry and replication within host macrophages.

### DUSP/Kinatase LINF_340027100 expression alters susceptibility to oxidative stress and trivalent antimony in *L. infantum* promastigotes

**Fig 4:**
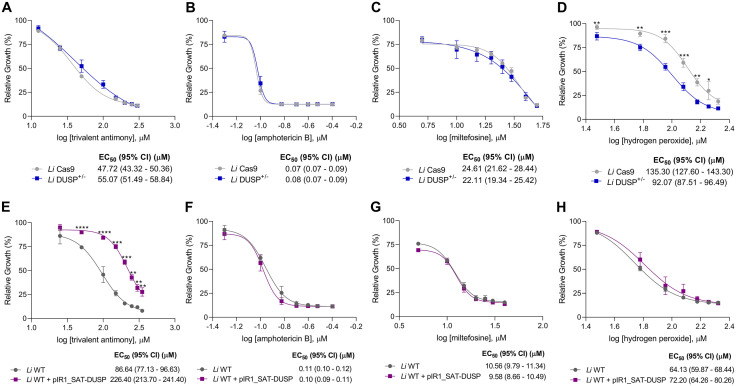
*In vitro* susceptibility analysis of DUSP/Kinatase LINF_340027100 mutant *L. infantum* promastigotes. Promastigotes parasites were cultured in the presence of different concentrations of **(A)** and **(E)** potassium antimony tartrate (Sb^III^) (25 to 350 μM), **(B)** and **(F)** amphotericin B (0.05 to 0.4 μM), **(C)** and **(G)** miltefosine (5 to 40 μM), and **(D)** and **(H)** hydrogen peroxide (30 to 210 μM). Parasite growth was determined after 48 h of incubation in the presence or absence of drugs. Data was plotted on dose-response curves in which the mean ± standard deviation of three independent experiments performed in triplicate are represented. The EC_50_ was determined using the linear interpolation method. Two-way ANOVA with Bonferroni’s post hoc test was applied to compare *Li* Cas9 and *Li* DUSP^+/-^, and *Li* WT and *Li* WT + pIR1_SAT-DUSP parasites for each drug concentration. * Represents significant differences in relation to the control (* *p* < 0.05, ** *p* < 0.01, *** *p* < 0.001 and **** *p* < 0.0001).

We evaluated the susceptibility of *L. infantum* promastigotes from *Li* Cas9 and *Li* DUSP^+/-^ mutants, as well as *Li* WT and *Li* WT + pIR1_SAT-DUSP parasites, to antileishmanial drugs, including trivalent antimony, amphotericin B, and miltefosine. In addition, we assessed the tolerance of these parasite lines to hydrogen peroxide, an inducer of oxidative stress (Fig 4).

Reduction of *DUSP* expression rendered the heterozygous knockout *Li* DUSP^+/-^ parasites approximately 1.5-fold more sensitive to hydrogen peroxide compared with the *L. infantum* Cas9-expressing control (EC_50_: 135.30 μM for *Li* Cas9 *vs*. 92.07 μM for *Li* DUSP^+/-^) (Fig 4D). In contrast, no significant differences in susceptibility were observed for trivalent antimony (EC_50_: 47.72 *vs.* 55.07 μM) (Fig 4A), amphotericin B (EC_50_: 0.07 μM *vs*. 0.08 μM) (Fig 4B), or miltefosine (EC_50_: 24.61 μM *vs.* 22.11 μM) (Fig 4C).

Conversely, *DUSP*-overexpressing parasites exhibited a 2.6-fold increase in resistance to trivalent antimony compared with *Li* WT parasites (EC_50_: 86.64 μM for *Li* WT vs. 226.40 μM for *Li* WT + pIR1_SAT-DUSP) (Fig 4E). No significant changes were detected in susceptibility to amphotericin B (EC_50_: 0.11 μM *vs.* 0.10 μM) (Fig 4F), miltefosine (EC_50_: 10.56 μM *vs.* 9.58 μM) (Fig 4G), or hydrogen peroxide (EC_50_: 64.13 μM *vs*. 72.20 μM) (Fig 4H).

Together, these findings suggest that *DUSP* acts as a key modulator of both redox balance and antimony susceptibility in *L. infantum*, with its overexpression promoting antimony resistance and its depletion increasing oxidative stress sensitivity.

### DUSP/Kinatase LINF_340027100 modulation affects oxidative stress response and antimony susceptibility in *L. infantum* intracellular amastigotes

**Fig 5:**
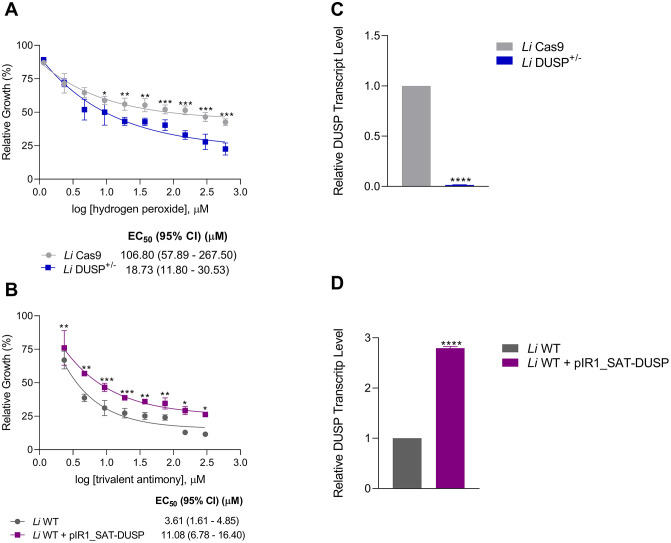
*In vitro* susceptibility analysis of DUSP/Kinatase LINF_340027100 mutant *L. infantum* intracellular amastigotes and quantification of DUSP transcript levels in axenic amastigote-like forms. The THP-1-derived macrophages were infected with the **(A)** *Li* Cas9 and *Li* DUSP^+/-^ and treated with hydrogen peroxide (1.2 to 600 µM) for 72 h; and **(B)** *Li* WT and *Li* WT + pIR1_SAT-DUSP and treated with trivalent antimony (2.3 to 300 µM) for 72 **h.** Data were plotted on dose-response curves in which the mean with standard deviations of three independent experiments performed in triplicate are represented. The EC_50_ was determined using the linear interpolation method. **(C)** Relative *DUSP* transcript level in *Li* DUSP^+/-^ axenic amastigotes-like forms. **(D)** Relative *DUSP* transcript level in *Li* WT + pIR1_SAT-DUSP axenic amastigotes-like. Relative *DUSP* gene transcript levels were determined by RT-qPCR. Relative quantification was performed using the 2^⁻ΔΔCt^ method, with the DNA polymerase gene used as an internal reference for normalization. Data represents the mean ± standard deviation of three independent experiments performed in triplicates. The T test was used to compare control parasite *Li* Cas9 and *Li* DUSP^+/-^ or *Li* WT and *Li* WT + pIR1_SAT-DUSP parasites. * Represents significant differences in relation to the control (* *p* < 0.05, ** *p* < 0.01, *** *p* < 0.001 and **** *p* < 0.0001).

Given that *Li* DUSP^+/-^ promastigotes exhibited increased sensitivity to hydrogen peroxide and *Li* WT + pIR1_SAT-DUSP parasites displayed enhanced resistance to trivalent antimony, we next examined whether these phenotypes were maintained in the intracellular amastigote stage. THP-1-derived macrophages were infected and subsequently exposed to serial concentrations of the respective compounds to determine EC_50_ values. Our results revealed that heterozygous knockout *Li* DUSP^+/-^ intracellular amastigotes were 5.7-fold more sensitive to hydrogen peroxide than *Li* Cas9 controls (EC_50_: 106.80 μM for *Li* Cas9 vs. 18.73 μM for *Li* DUSP^+/-^) (Fig 5A). Conversely, *DUSP*-overexpressing intracellular amastigotes exhibited a 3.1-fold increase in resistance to trivalent antimony compared with *Li* WT parasites (EC_50_: 3.61 μM for *Li* WT vs. 11.08 μM for *Li* WT + pIR1_SAT-DUSP) (Fig 5B). These findings suggest that *DUSP* expression has an even greater impact on parasite susceptibility during the intracellular amastigote stage, reinforcing its potential role in modulating redox balance and antimony tolerance within the host environment.

Given the pronounced phenotypes observed in intracellular amastigotes, we next examined *DUSP* expression in axenic amastigote-like forms derived from the *L. infantum* lines. cDNA was prepared from axenic amastigote-like parasites to quantify *DUSP* transcript levels. The heterozygous knockout *Li* DUSP^+/−^ line exhibited a 71.4-fold reduction in transcript abundance (Fig 5C) compared with *Li* Cas9 controls. Conversely, *DUSP*-overexpressing amastigote-like forms displayed a 2.8-fold increase in transcript levels (Fig 5D) relative to *Li* WT parasites. These results indicate that *DUSP* expression is stage-regulated and support an important role for this phosphatase in maintaining amastigote physiology and adaptation to the intracellular environment.

### Modulation of DUSP/Kinatase LINF_340027100 affects the transcriptional regulation of *MAPK1*, *MAPK3*, and *MAPK10* in *L. infantum*

**Fig 6:**
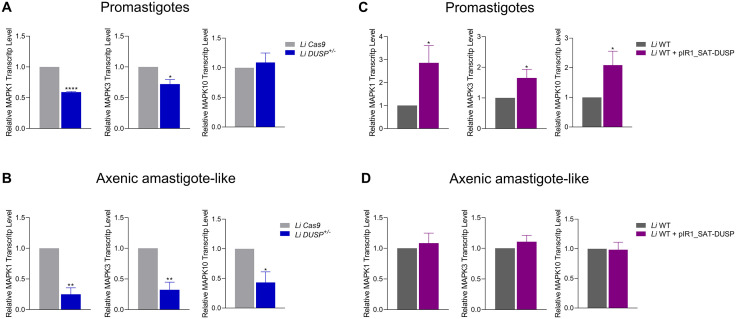
Analysis of *MAPK1*, *MAPK3*, and *MAPK10* transcript levels in promastigotes and axenic amastigote-like forms of *Li* Cas9, *Li* DUSP^+^^/-^, *Li* WT and *Li* WT + pIR1_SAT-DUSP. Relative *MAPK1*, *MAPK3* and *MAPK10* transcript level in *DUSP* heterozygous knockout **(A)** promastigote and **(B)** axenic amastigote-like forms, and in *DUSP* overexpressing **(C)** promastigote and **(D)** axenic amastigote-like forms. Relative transcript levels were determined by RT-qPCR. Relative quantification was performed using the 2^⁻ΔΔCt^ method, with the DNA polymerase gene used as an internal reference for normalization. The T test was used to compare control parasite *Li* Cas9 and *Li* DUSP^+/-^ or *Li* WT and *Li* WT + pIR1_SAT-DUSP parasites. Data represents the mean ± standard deviation of three independent experiments performed in triplicates. T test was used to compare control parasite *Li* Cas9 and *Li* DUSP^+/-^ or *Li* WT and *Li* WT + pIR1_SAT-DUSP parasites. *Represents significant differences in relation to the control parasite (* *p* < 0.05, ** *p* < 0.01 and **** *p* < 0.0001).

To investigate whether modulation of *DUSP* expression affects MAPK-mediated signaling in *L. infantum*, we analyzed the transcript levels of three representative members of this kinase family — *MAPK1*, *MAPK3*, and *MAPK10* (Fig 6). These isoforms were selected because they represent distinct, yet complementary biological roles previously characterized in *Leishmania*: MAPK1 is essential for parasite survival and trivalent antimony resistance [26–28]; MAPK3 regulates flagellar biogenesis and infectivity [29] and *MAPK10* contributes intracellular adaptation [30,31].

Together, their evaluation provides an integrated view of how *DUSP* modulation may influence the overall dynamics of MAPK signaling. Transcript expression levels were quantified by RT-qPCR in both promastigote and axenic amastigote-like stages of the *DUSP* heterozygous knockout and *DUSP*-overexpressing *L. infantum* lines, along with their respective control lines. Because MAPK activation primarily occurs via post-translational phosphorylation, transcriptional profiling was used as an indirect approach to infer regulatory adjustments in MAPK signaling, potentially reflecting compensatory responses to altered *DUSP* expression.

In heterozygous knockout *Li* DUSP *+ / −* promastigotes, transcript levels of *MAPK1* and *MAPK3* were reduced by 1.7- and 1.4-fold, respectively, whereas *MAPK10* expression remained comparable to that of the *Li* Cas9 control (Fig 6A). In contrast, in the axenic amastigote-like stage, all three MAPKs exhibited marked transcriptional downregulation, with approximately 4.0-, 3.1-, and 2.3-fold reductions in *MAPK1*, *MAPK3*, and *MAPK10* transcript levels, respectively (Fig 6B). These results indicate a broad perturbation of MAPK signaling homeostasis in the *DUSP* heterozygous knockout line. Collectively, these findings suggest that partial depletion of *DUSP* disrupts the regulatory balance of kinase signaling, potentially triggering compensatory feedback mechanisms that adjust MAPK gene expression in response to altered phosphorylation dynamics.

Conversely, *DUSP*-overexpressing promastigotes exhibited significant upregulation of *MAPK1*, *MAPK3*, and *MAPK10* transcripts, with increases of 2.9, 1.7, and 2.1-fold, respectively (Fig 6C). This pattern suggests a compensatory transcriptional response to *DUSP* overexpression aimed at restoring signaling equilibrium. In contrast, no significant changes in *MAPK* transcript levels were detected in the axenic amastigote-like stage (Fig 6D), indicating that stage-specific regulatory mechanisms may limit the transcriptional compensation observed in promastigotes.

## Discussion

Leishmaniasis remains a major global health burden, highlighting the need to identify novel molecular targets given the limited number of available chemotherapeutic agents, their toxicity, prolonged treatment regimens, and the emergence of drug-resistant parasite strains [2,3]. In this context, the functional characterization of signaling regulators, particularly phosphatases, provides important insights into the molecular mechanisms governing parasite adaptation and survival. Phosphatases are key components of *Leishmania* signaling networks, acting as modulators of phosphorylation-dependent pathways that regulate differentiation, stress responses, and host–parasite interactions [7,9,10–13]. Among them, dual-specificity phosphatases (DUSPs) are notable for their ability to dephosphorylate both tyrosine and serine/threonine residues, thereby regulating mitogen-activated protein kinase (MAPK) signaling pathways that control proliferation, differentiation, and responses to oxidative stress in *Leishmania* [8,10–12]. In this study, we provide the first functional characterization of the atypical dual-specificity phosphatase/kinatase DUSP (LINF_340027100) in *L. infantum*, demonstrating that this protein modulates infectivity, oxidative stress response, differentiation, and antimony susceptibility.

Attempts to generate null mutants using the CRISPR/Cas9 system were unsuccessful, as no viable parasites were recovered after disruption of both alleles, suggesting that *DUSP* is essential for promastigote viability. A complementary strategy involving episomal expression of *DUSP* prior to chromosomal deletion, similar to that used for the ascorbate peroxidase gene [32], yielded viable transfectants but failed to eliminate the endogenous locus. The persistence of the chromosomal allele may reflect genomic plasticity mechanisms such as aneuploidy or gene amplification, which are well-documented adaptive strategies in *Leishmania* that preserve essential gene functions under selective pressure [33–36]. These findings suggest that DUSP/Kinatase performs an important cellular function in *L. infantum*.

To investigate the functional relevance of DUSP/Kinatase, we generated *L. infantum* lines with experimentally modulated expression levels. Partial reduction of *DUSP* expression resulted in pronounced alterations in cell morphology and cell-cycle distribution without substantially affecting overall growth kinetics, suggesting that compensatory mechanisms may partially sustain proliferation despite perturbation of signaling balance. *DUSP*-deficient parasites displayed an elongated and slender morphology with increased flagellar length, indicating disruption of cytoskeletal organization, a process tightly regulated by phosphorylation-dependent pathways in trypanosomatids. In parallel, the altered distribution of cells across cell-cycle phases, characterized by reduced representation in G0/G1 and accumulation in S phase, supports the hypothesis that *DUSP* contributes to the coordination of regulatory checkpoints controlling DNA replication and cell division.

Conversely, increased *DUSP* expression produced a distinct phenotype characterized by reduced cell size, rounded morphology, shorter flagella, and modest impairment of proliferation. The enrichment of parasites in S phase under both reduced and elevated *DUSP* expression suggests that perturbation of kinase–phosphatase equilibrium interferes with the timely progression of DNA synthesis, possibly reflecting replication-associated stress. Together, these findings indicate that balanced *DUSP* expression is required to maintain promastigote morphology, flagellar architecture, and proper cell-cycle dynamics. Comparable defects in morphology and proliferation have been reported following genetic or pharmacological disruption of protein kinases in *Leishmania*, highlighting the importance of tightly regulated kinase–phosphatase signaling networks for parasite development and proliferative fitness [37,38]. Additionally, both *DUSP*-heterozygous and *DUSP*-overexpressing parasites exhibited a reduced proportion of metacyclic forms, indicating that precise regulation of *DUSP* expression is required for efficient metacyclogenesis. These findings suggest that DUSP functions as a fine-tuning regulator of parasite differentiation, as both decreased and increased expression impair the generation of infective forms.

*DUSP*-modulated lines also displayed reduced intracellular proliferation during macrophage infection. Reduced intracellular replication was observed for both *Li* DUSP^+/-^ and *DUSP*-overexpressing parasites relative to *Li* WT controls. This phenotype is likely driven by impaired metacyclogenesis, as the lower amastigote numbers persist despite equal or higher host cell entry—indicating that active infection cannot be established as successfully. It was already demonstrated that metacyclic forms have a markedly higher survival rate in macrophages compared to non-metacyclic forms due to distinct entry pathways and phagolysosome modulation [39].

In addition, the lower infectivity could also indicate that precise regulation of this phosphatase is required for parasite fitness within host cells. The amastigote stage relies on efficient antioxidant defenses to counteract host-derived reactive oxygen species [40,41], suggesting that altered *DUSP* expression may impair the parasite’s ability to respond to oxidative stress. Consistent with this hypothesis, the *Li* DUSP^+/-^ line exhibited increased sensitivity to hydrogen peroxide, whereas *DUSP*-overexpressing parasites displayed enhanced resistance to trivalent antimony (Sb^III^). Antimony resistance in *Leishmania* has been associated with mechanisms such as altered thiol metabolism, decreased drug accumulation, and increased efflux mediated by ABC transporters including MRPA [3,42,43]. Notably, *DUSP* transcripts have previously been reported to be upregulated in Sb^III^-resistant *L. infantum* lines [14], supporting a potential role for this phosphatase in drug response pathways.

MAPK signalling pathways are central regulators of *Leishmania* adaptation and virulence. *MAPK1*, *MAPK3*, and *MAPK10* have been implicated in parasite proliferation, differentiation, and stress responses [26–31,44–46]. Because DUSPs function as negative regulators of MAPK activity, modulation of *DUSP* expression is expected to influence MAPK signaling dynamics. Consistent with this idea, transcriptional analyses revealed downregulation of *MAPK1* and *MAPK3* in the *Li* DUSP^+/-^ line, whereas the overexpressing parasites displayed increased *MAPK* transcript levels in promastigotes. These transcriptional changes likely reflect compensatory feedback mechanisms aimed at maintaining signalling homeostasis. Importantly, MAPK activity is primarily regulated through phosphorylation rather than transcription, and therefore the observed alterations probably represent indirect responses to changes in signalling equilibrium rather than direct regulation of MAPK enzymatic activity. Further investigations, including phosphoproteomic and functional assays, will be required to elucidate the precise mechanisms underlying this regulatory interplay.

In summary, our findings identify the atypical dual-specificity phosphatase/kinatase DUSP as a key regulator of *L. infantum* biology. Modulation of *DUSP* expression demonstrates that tightly controlled phosphatase activity is required to maintain parasite morphology, cell-cycle progression, differentiation, and adaptive responses to oxidative stress and antimonial pressure. These results underscore the importance of balanced kinase–phosphatase signalling for preserving cellular homeostasis and developmental competence in *Leishmania*. Overall, this study provides new insights into MAPK-associated regulatory networks in trypanosomatids and highlights DUSP as a promising candidate for therapeutic exploration, particularly in the context of parasite adaptation and drug resistance.

## Supporting information

S1 Table List of primers used in this study. (PDF)

S2 Table Complete growth curve dataset for *DUSP* mutant *L. infantum* lines. An initial inoculum of 1 x 10^5^ promastigote forms per mL was prepared for the *Li* Cas9, *Li* DUSP^+/-^, *Li* WT and *Li* WT + pIR1_SAT-DUSP parasites, which were counted every 24 h using the Z1 Coulter Counter. The data are presented as the mean and standard deviation of three experiments performed in triplicate. Two-way ANOVA with Bonferroni’s post hoc test was used to compare *Li* Cas9 and *Li* DUSP^+/-^, and *Li* WT and *Li* WT + pIR1_SAT-DUSP parasites at each time point. * Represents significant differences in relation to the control parasite (* *p* < 0.05).
(PDF)

S1 Fig Multiple sequence alignment of putative dual-specificity phosphatase/kinatase protein sequences from *Leishmania infantum* (LINF_340027100-T1) and two *Leishmania major* sequences (CAG95820.1 and LmjF.34.2190). Amino acid sequences were aligned using CLUSTAL O (version 1.2.3). The two *L. major* sequences correspond to entries derived from the Friedlin strain, obtained from distinct genome sequencing projects and annotation pipelines.
(PDF)

S2 Fig Structural and domain architecture of LINF_340027100. (A) Schematic representation of the predicted domain structure of DUSP. Domain boundaries and annotations were obtained from PFAM (via TriTrypDB release 68, May 2024). (B) Predicted three-dimensional structure of DUSP generated by AlphaFold (https://alphafold.ebi.ac.uk/entry/A4I9Z1). The protein is color-coded according to the per-residue confidence score (pLDDT), where blue indicates high confidence and orange indicates low confidence. (C) AlphaFold-predicted structure of DUSP colored by domain.
(TIF)

S3 Fig Characterization knockout of *DUSP* endogenous gene in *L*. *infantum* expressing *L. infantum DUSP* epissomally. (A) The integration of the resistance marker neomycin (944 bp) and (B) puromycin (854 bp) was evaluated by PCR by annealing a primer in a 5′UTR region adjacent to the cassette (primer P1) and another primer annealed within resistance marker sequence (primer P2 and P6, respectively). (C) Fragment *DUSP*-coding sequence was amplified using PCR with a primer in a 5′UTR region adjacent to the cassette (primer P1) and primer P4. MW: molecular weight; NC: negative control; bp: base pair.
(TIF)

S4 Fig Representative histograms of cell cycle analysis of *DUSP* mutants. Cell cycle analysis of (A) and (B) *DUSP* heterozygous knockout at 48 hours and 96 hours, respectively. (C) and (D) cell cycle analysis of *Li* WT and *Li* WT + pIR1_SAT-DUSP parasites at 48 hours and 96 hours, respectively. The parasites were stained with propidium iodide and evaluation by flow cytometry.
(TIF)
