# Supplementary material for: The atypical dual-specificity protein phosphatase (DUSP)/kinatase of Leishmania infantum modulates infectivity, oxidative stress response and antimonial resistance
Source: PLoS Negl Trop Dis. 2026 May 26;20(5):e0014330. doi: 10.1371/journal.pntd.0014330 (PMC13210143; doi:10.1371/journal.pntd.0014330)
Supplement: S1 Table — (PDF) [file pntd.0014330.s001.pdf]

S1 Table. List of primers used in this study.

PRIMER NAME	PRIMER SEQUENCE 5’ → 3’
**LiDUSP_DNADoador_F1**	CATGGCACGTCGCGCGAAGCCTCCGTCGCAgtataatgcagacctgctgc
**LiDUSP_DNADoador_R5**	CAAGAAGACAGGACCAGCGAATACGCATACccaatttgagagacctgtgc
**sgRNA_Rv**	AAAAGCACCGACTCGGTGCCACTTTTTCAAGTTGATAACGGACTAGCCTTATTTTAACTTGCTATT TCTAGCTCTAAAAC
**LiDUSP_5'sgRNA_Fw**	gaaattaatacgactcactataggGCGCGGGCGGACAACTTCTGgttttagagctagaaatagc
**LiDUSP_3'sgRNA_Fw**	gaaattaatacgactcactataggTGCGCCTATGTGCACGTGAGgttttagagctagaaatagc
**LiDUSP_Int_Fw (P3)**	GAGGACGCGATCAGACTCTG
**LiDUSP_Int_Rv (P4)**	ACATGTAGTCCTGCACCACG
**NEO_Rv (P2)**	GCCAACGCTATGTCCTGATA
**PURO_Rv (P6)**	GGCTTACGTGTCATGCACCAT
**LiDUSP_5’UTR_Fw (P1)**	GTGGAGCACGGCAAGGTAC
**LiDUSP_RTqPCR_Fw**	ACAGCCGTGCGAAAACAAAG
**LiDUSP_RTqPCR_Rv**	AGTTTTGCGCGAAACTCAGG
**RTqPCR_DNApol_Fw**	CGAGGGCAAGACATAC
**RTqPCR_DNApol_Rv**	GAGAGCGGGCACCAATCAC
**GibsonAssembly_DUSP_Fw**	cctcgctgcccgcgtccggagatctATGTGCGAGGTGCTCGAC
**GibsonAssembly_DUSP_Rv**	agtacatcacaagactcatagatctTCAGTTTGCTGAACGCCC
**MAPK1_Fw**	GAACATCATTCACCGGGATTTG
**MAPK1_Rv**	GTAGTCAGTCAGGTCGAGAAAC
**MAPK3_Fw**	CGCTGGAGAAGCTGAATGAA
**MAPK3_Rv**	CGTCCACATCTTGGAGAGAAAG
**MAPK10_Fw**	TCATCTCGCCACAGCATATC
**MAPK10_Rv**	GATGTCGTTGTTGTCTGCTAAC
