# Supplementary material for: The atypical dual-specificity protein phosphatase (DUSP)/kinatase of Leishmania infantum modulates infectivity, oxidative stress response and antimonial resistance
Source: PLoS Negl Trop Dis. 2026 May 26;20(5):e0014330. doi: 10.1371/journal.pntd.0014330 (PMC13210143; doi:10.1371/journal.pntd.0014330)
Supplement: S2 Table — An initial inoculum of 1 x 10^5^ promastigote forms per mL was prepared for the Li Cas9, Li DUSP+/-, Li WT and Li WT + pIR1_SAT-DUSP parasites, which were counted every 24 h using the Z1 Coulter Counter. The data are presented as the mean and standard deviation of three experiments performed in triplicate. Two-way ANOVA with Bonferroni’s post hoc test was used to compare Li Cas9 and Li DUSP+/-, and Li WT and Li WT + pIR1_SAT-DUSP parasites at each time point. * Represents significant differences in relation to the control parasite (* p < 0.05). (PDF) [file pntd.0014330.s002.pdf]

S2 Table. Complete growth curve dataset for *DUSP* mutant *L. infantum* lines.

	Number of parasites (x10^5/mL)	
**Time (hours)**	***Li* WT**	***Li* WT + pIR1_SAT-DUSP**
0	1	1
24	3.6 ± 0.2	5.1 ± 0.1
48*	24.1 ± 0.9	16.2 ± 3.5
72*	123.1 ± 4.4	62.2 ± 13.9
96	379.3 ± 73.0	287.4 ± 30.9
120	396.8 ± 66.8	424.4 ± 78.9
144	495.4 ± 40.1	648.8 ± 247.1
168	396.0 ± 55.0	484.5 ± 74.3

	Number of parasites (x10^5/mL)	
**Time (hours)**	***Li* Cas9**	***Li* DUSP$^{+/-}$**
0	1	1
24	1.9 ± 0.5	2.0 ± 0.7
48	7.1 ± 1.4	8.8 ± 2.1
72	57.8 ± 1.0	51.5 ± 5.9
96	228.2 ± 12.6	224.8 ± 9.2
120	799.8 ± 57.3	871.3 ± 39.1
144	949.9 ± 35.9	998.0 ± 40.9
168	952.2 ± 95.3	867.4 ± 54.2
