# Supplementary material for: The atypical dual-specificity protein phosphatase (DUSP)/kinatase of Leishmania infantum modulates infectivity, oxidative stress response and antimonial resistance
Source: PLoS Negl Trop Dis. 2026 May 26;20(5):e0014330. doi: 10.1371/journal.pntd.0014330 (PMC13210143; doi:10.1371/journal.pntd.0014330)
Supplement: S1 Fig — Amino acid sequences were aligned using CLUSTAL O (version 1.2.3). The two L. major sequences correspond to entries derived from the Friedlin strain, obtained from distinct genome sequencing projects and annotation pipelines. (PDF) [file pntd.0014330.s003.pdf]

```
LINF_340027100-T1-p1      ------------------------------------------------------------  0
CAG9582091.1              ------------------------------------------------------------  0
LmjF.34.2190:mRNA-p1      MGVLCGSANSHGGRILAGCADTNCRSNQELSRCRVDSTHAKRNLCAQHSHFSSSAEVGDA  60


LINF_340027100-T1-p1      ------------------------------------------------------------  0
CAG9582091.1              ------------------------------------------------------------  0
LmjF.34.2190:mRNA-p1      NLSETILSASTIHISRVGGSGRSKRLASVGDDTNSDGVGHLSAVGAAARGAAEHGKLLQA  120


LINF_340027100-T1-p1      ------------------------------------------------------------  0
CAG9582091.1              ------------------------------------------------------------  0
LmjF.34.2190:mRNA-p1      PGTAQPTLQSPHAAADAAQNGHEQQQLQRGASSHGTLREASVAPQKLSARALRIVSRVDS  180


LINF_340027100-T1-p1      -------------------MCEVLDALRSSSVGLLFLNHPEVSFSNRSCVICIPDFHQDE  41
CAG9582091.1              -------------------MYEVLDALRSSSVGLLFRNHPEVSFSNRSCVICIPDFHQDE  41
LmjF.34.2190:mRNA-p1      IVRKCEGLDAERPTEEEKVMYEVLDALRSSSVGLLFRNHPEVSFSNRSCVICIPDFHQDE  240
                                             * *************** ***********************

LINF_340027100-T1-p1      TIDGYRLRAAYNAGTIGRSYFATRDVAALSSRCTPPHGSSDSLSDDAARGPVFRILKVIS  101
CAG9582091.1              TIDGYRLRAAYNAGTIGRSYFATRDIAALSSRCTPPHGSSDCVSDDAGKGPVFRILKVIS  101
LmjF.34.2190:mRNA-p1      TIDGYRLRAAYNAGTIGRSYFATRDIAALSSRCTPPHGSSDCVSDDAGKGPVFRILKVIS  300
                          *************************:***************.:****.:***********

LINF_340027100-T1-p1      FVLRRRLLEEITAEQRVLVNIVHQNVLQISDVLNDEAKENMIVITNYHAKGNIGNYAGRL  161
CAG9582091.1              FVLRRRLLEEITAEQRVLVNIVHQNVLHISDVLNDEAKENMIVITNYHAKGNIGNYAGRL  161
LmjF.34.2190:mRNA-p1      FVLRRRLLEEITAEQRVLVNIVHQNVLHISDVLNDEAKENMIVITNYHAKGNIGNYAGRL  360
                          ***************************:********************************

LINF_340027100-T1-p1      SHDSDKLRRILTEVAVGLRILHSHRVYHHNLKLDNVLENGEGHFCIADAGFWRLFAVQCP  221
CAG9582091.1              SHDSDKLRRILVEVAVGLRILHSHRVYHHNLKLDNVLENSEGHFCIADAGFWRLFAVQCP  221
LmjF.34.2190:mRNA-p1      SHDSDKLRRILVEVAVGLRILHSHRVYHHNLKLDNVLENSEGHFCIADAGFWRLFAVQCP  420
                          ***********.***************************.********************

LINF_340027100-T1-p1      EDLVFNGELACLPPEVFDPEGPYATGEVNVVSDEGAAGVAAVDIWGFGVLMYRLAYGCDP  281
CAG9582091.1              EDLVFNGELACLPPEVFDPEGPYATGEVNVVSDEGAAGVAAVDIWGFGVLMYRLAYGCDP  281
LmjF.34.2190:mRNA-p1      EDLVFNGELACLPPEVFDPEGPYATGEVNVVSDEGAAGVAAVDIWGFGVLMYRLAYGCDP  480
                          ************************************************************

LINF_340027100-T1-p1      IEIAECSYAQVHERLMGFDLSFPPRPHWSFAYDIEDAIRLCLQKVPSKRPSVLRLLQHPF  341
CAG9582091.1              VEIAECSYAQVHERLMGFDLSFPPRPHWSFAYDIEDAIRLCLQKEPSKRPSVLRLLQHTF  341
LmjF.34.2190:mRNA-p1      VEIAECSYAQVHERLMGFDLSFPPRPHWSFAYDIEDAIRLCLQKEPSKRPSVLRLLQHTF  540
                          :******************************************* ************* *

LINF_340027100-T1-p1      FKHSLVVGTSSLMRKMSMTSSFAFGGQMVGSFGDRTMSAPGLRGASMSSMAVPNYRTQQR  401
CAG9582091.1              FKHSLVVGTSSLMRKMSMTSSFAFGGQMVGSFGDQTMSALALRGASISSVAVPNYRTQQR  401
LmjF.34.2190:mRNA-p1      FKHSLVVGTSSLMRKMSMTSSFAFGGQMVGSFGDQTMSALALRGASISSVAVPNYRTQQR  600
                          **********************************:**** .*****:**:**********

LINF_340027100-T1-p1      NGFQVDAFLGEGRFSETMMVHLRRNHSKQFAFKIIYKSILKRLQAPGREKWAREMRRQLV  461
CAG9582091.1              NGFQVDAFLGEGRFSETMMVHLRRNHSKQFAFKIIYKSILRRLQAPGRERWAREMRRQLV  461
LmjF.34.2190:mRNA-p1      NGFQVDAFLGEGRFSETMMVHLRRNHSKQFAFKIIYKSILRRLQAPGRERWAREMRRQLV  660
                          ****************************************:********:**********

LINF_340027100-T1-p1      FSRKVDHPNVMRFIDIVEDKKVNCFVVQDYMSGGSIEAVPPVKGDSSSPTLQDFLVDVLA  521
CAG9582091.1              FSRKVDHPNVMRFIDIVEDKKVNCFVVQDYMSGGAIEAVPPVKGDSSSPTLQEFLVDVLA  521
LmjF.34.2190:mRNA-p1      FSRKVDHPNVMRFIDIVEDKKVNCFVVQDYMSGGAIEAVPPVKGDSSSPTLQEFLVDVLA  720
                          **********************************:*****************:*******

LINF_340027100-T1-p1      GLVHLHDNGVAHLSLLPTNIFFCEHTLHYCIADFGPLFVTADALADSIAEGAPLYALPAW  581
CAG9582091.1              GLVHLHDNGVAHLSLLPTNIFFCEHTFHYRIADFGPLFVTADTLVDSIAEGAPLYRLPAW  581
LmjF.34.2190:mRNA-p1      GLVHLHDNGVAHLSLLPTNIFFCEHTFHYRIADFGPLFVTADTLVDSIAEGAPLYRLPAW  780
                          **************************:** ************:*.********** ****

LINF_340027100-T1-p1      VRRHSPLHGPSVDMFCVGLLAASVLPELFDTVWAELLDSEKNRTFAVDAVLTAVRKQRAQ  641
CAG9582091.1              VQRHSPLHGPGVDMFCVGLLAASVLPELFSTVWAELLDGEKSKTFAVEKVLTAVQKSRAQ  641
LmjF.34.2190:mRNA-p1      VQRHSPLHGPGVDMFCVGLLAASVLPELFSTVWAELLDGEKSKTFAVEKVLTAVQKSRAQ  840
                          *:********.******************.********.**.:****: *****:*.***

LINF_340027100-T1-p1      LTPALVSFIEDALEGRFQDARAALKHTYFGNLSFAQNLPKTIVEVTEEELQSAVHSKPET  701
CAG9582091.1              LTPALISFIEDALEGRFEDARAALKHTYFRNLSFAQNLPKTIVEVTEEELQSAVHSKPET  701
LmjF.34.2190:mRNA-p1      LTPALISFIEDALEGRFEDARAALKHTYFRNLSFAQNLPKTIVEVTEEELQSAVHSKPET  900
                          *****:***********:*********** ******************************

LINF_340027100-T1-p1      RDEARMLEVLAQDPFQESQMLSSAGDATLHGSESCTEASVIAGATGEKPTVLVFEGENLL  761
CAG9582091.1              RDEARMLEVLAQDPFQESQMLSSAGDATLHGSESCTEASVIAVAAGEKPTVLVFQGENLL  761
LmjF.34.2190:mRNA-p1      RDEARMLEVLAQDPFQESQMLSSAGDATLHGSESCTEASVIAVAAGEKPTVLVFQGENLL  960
                          ****************************************** *:*********:*****

LINF_340027100-T1-p1      CGQCSAELTVALYQCSDCDGYIRCGKCSVGNYHKDGHELVPFLIHTIEHSRDGANKAVLV  821
```

```
CAG9582091.1              CGQCSAELTVALYQCSDCDSYIRCGKCSVGNYHKDGHELVPLLIHTIEHSRDGANKAVLV   821
LmjF.34.2190:mRNA-p1      CGQCSAELTVALYQCSDCDSYIRCGKCSVGNYHKDGHELVPLLIHTIEHSRDGANKAVLV   1020
                          ******************.*************:***************************

LINF_340027100-T1-p1      QPSTVPDVHALETLEMTANFPVGSLTAHLVAQRAAERSIAVRSTGGGSITKGMFGDMESV   881
CAG9582091.1              QPSTVPDVHALETLEMTANFPVGSLTAHLVAQRAAERSIAVRSTGGGSITKGMFGDMESV   881
LmjF.34.2190:mRNA-p1      QPSTVPDVHALETLEMTANFPVGSLTAHLVAQRAAERSIAVRSTGGGSITKGMFGDMESV   1080
                          ************************************************************

LINF_340027100-T1-p1      VRLPDDISEQSISVNINCRSFISFRGLGGMPGGGLNLGSGANNSNTGFNTVGFPHGRADP   941
CAG9582091.1              VRLPDDISEQSISVNINGRSFISFRGLGGVPGGGLNLGSGANNSNTGFSTAGFPNGRADA   941
LmjF.34.2190:mRNA-p1      VRLPDDISEQSISVNINGRSFISFRGLGGVPGGGLNLGSGANNSNTGFSTAGFPNGRADA   1140
                          ***************** ***********:******************.*.***:****

LINF_340027100-T1-p1      VTPKDFGSLCLPPPTRLSLMKGKEARKLVLPKAEEIEDDDWQQELERCRTSNHSELLLYN   1001
CAG9582091.1              VTPKDSGSLCLPPPMRLSLMKDKEARKLALPKAEEIEDDDWQQELERCRTSNHSELLLYN   1001
LmjF.34.2190:mRNA-p1      VTPKDSGSLCLPPPMRLSLMKDKEARKLALPKAEEIEDDDWQQELERCRTSNHSELLLYN   1200
                          ***** ******** ******.******.*******************************

LINF_340027100-T1-p1      YGLDEVPPEVYDPPLLQVVVLDISQNNLRSLPHELSFLIHLRKLVVSYNKLTELPDSLGN   1061
CAG9582091.1              YGLDEVPPEVYDPPLLQVVILDISQNNLRSLPHELSFLIHLRKLVVSYNKLTELPDSLGN   1061
LmjF.34.2190:mRNA-p1      YGLDEVPPEVYDPPLLQVVILDISQNNLRSLPHELSFLIHLRKLVVSYNKLTELPDSLGN   1260
                          *******************:****************************************

LINF_340027100-T1-p1      LSELESLDASHNALVDLPQTFIYLSSLTSAALDYNNFSSIPDSLLDIVAPPLCSSASNVM   1121
CAG9582091.1              LSELESLDASHNALVDLPQTFIYLSSLTSAALDYNSFSSIPDSLLDIVAPPLCSSASNVM   1121
LmjF.34.2190:mRNA-p1      LSELESLDASHNALVDLPQTFIYLSSLTSAALDYNSFSSIPDSLLDIVAPPLCSSASNVM   1320
                          ***********************************.************************

LINF_340027100-T1-p1      ENFAMSTTQVNGARMASLVGNTAVSLVGGSSVSNSKTVIMSPKLKVIYLAANDSLTTLPL   1181
CAG9582091.1              ENFTMATPQVNGTRIASFMFNPAGSLVGGSSVSNSKAVIMSPKLKVIYLAANDSLTTLPL   1181
LmjF.34.2190:mRNA-p1      ENFTMATPQVNGTRIASFMFNPAGSLVGGSSVSNSKAVIMSPKLKVIYLAANDSLTTLPL   1380
                          ***:*:* ****:*:**:: * * ************:***********************

LINF_340027100-T1-p1      RERLQRFDDLTIALDNEPSLYKDYYEKNLDTELPNITVNWNKLYPDEIVPYLYCGSLRSA   1241
CAG9582091.1              RERLQRFDDLTIALDNEPSLYKDYYEKNLDTELPNITVNWNKLYPDEIVPYLYCGSLRSA   1241
LmjF.34.2190:mRNA-p1      RERLQRFDDLTIALDNEPSLYKDYYEKNLDTELPNITVNWNKLYPDEIVPYLYCGSLRSA   1440
                          ************************************************************

LINF_340027100-T1-p1      QSQMVYRKLNITYLLTVGRQLVPVPPEGGHHKVIVVDDIPGANIRMSFQEAVNFIEESQS   1301
CAG9582091.1              QSQMVYRKLNITYLLTVGRQLVPVPPEGGHHKIIVVDDIPGANIRMSFQEAVDFIEESQS   1301
LmjF.34.2190:mRNA-p1      QSQMVYRKLNITYLLTVGRQLVPVPPEGGHHKIIVVDDIPGANIRMSFQEAVDFIEESQS   1500
                          ********************************:*******************:*******

LINF_340027100-T1-p1      KKSGCLVHCFAGLSRSATTVIAYLMIKRGMRLDEAYLVTKKGRPAILPNKGFFDQLVELD   1361
CAG9582091.1              KKSGCLVHCFAGLSRSATTVIAYLMIKRGMRLDEAYRVTKKGRPAILPNKGFFDQLVELD   1361
LmjF.34.2190:mRNA-p1      KKSGCLVHCFAGLSRSATTVIAYLMIKRGMRLDEAYRVTKKGRPAILPNKGFFDQLVELD   1560
                          ************************************ ***********************

LINF_340027100-T1-p1      KELYPKPNRPLDIESLGRSAN     1382
CAG9582091.1              KELYPKPDRPLDIESLGRSAN     1382
LmjF.34.2190:mRNA-p1      KELYPKPDRPLDIESLGRSAN     1581
                          *******:*************
```

S1 Fig. Multiple sequence alignment of putative dual-specificity phosphatase/kinatase protein sequences from *Leishmania infantum* (LINF_340027100-T1) and two *Leishmania major* sequences (CAG95820.1 and LmjF.34.2190). Amino acid sequences were aligned using CLUSTAL O (version 1.2.3). The two *L. major* sequences correspond to entries derived from the Friedlin strain, obtained from distinct genome sequencing projects and annotation pipelines.
