# Supplementary figures and images for: The atypical dual-specificity protein phosphatase (DUSP)/kinatase of Leishmania infantum modulates infectivity, oxidative stress response and antimonial resistance
Source: PLoS Negl Trop Dis. 2026 May 26;20(5):e0014330. doi: 10.1371/journal.pntd.0014330 (PMC13210143; doi:10.1371/journal.pntd.0014330)

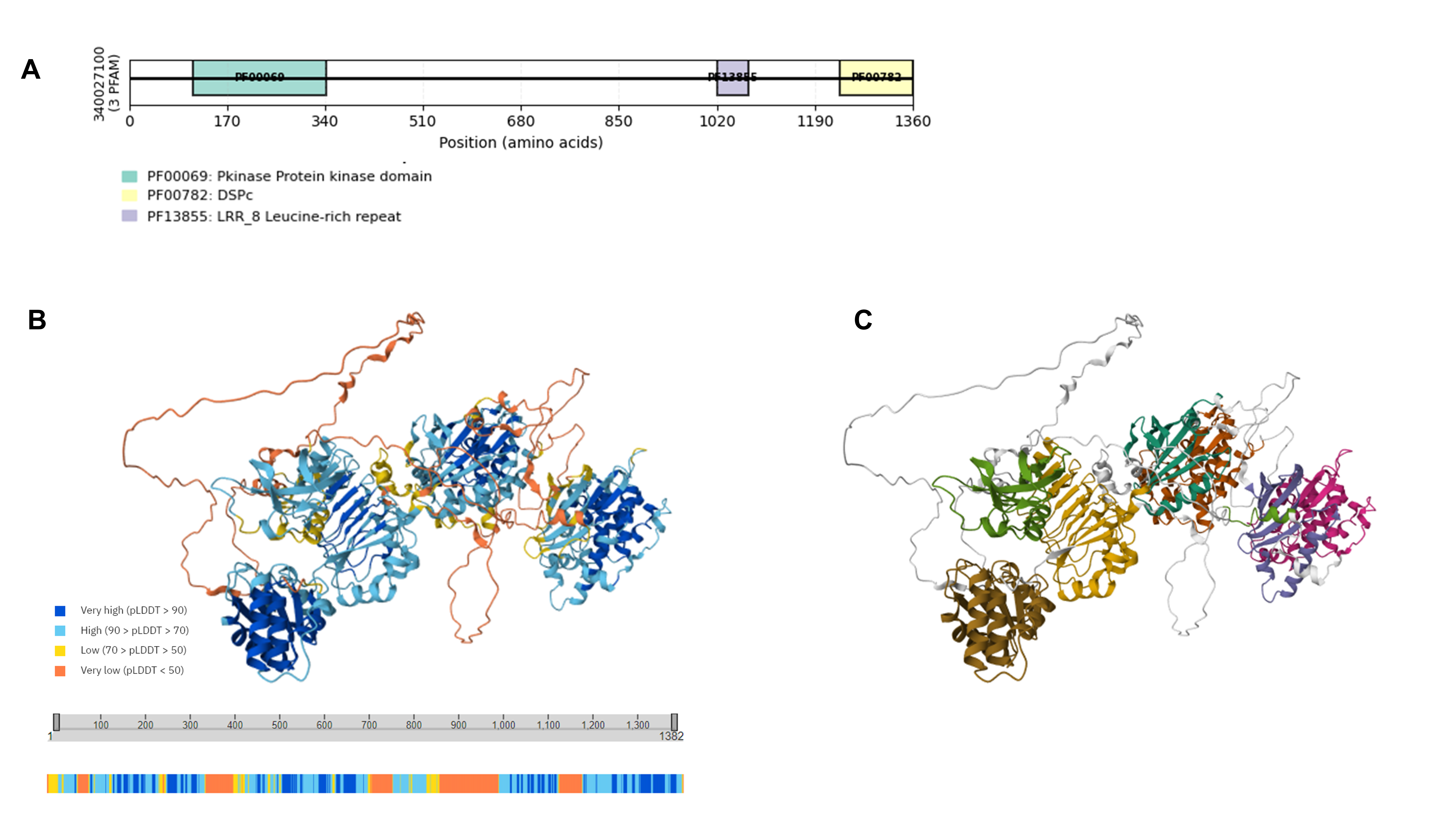

Supplement: S2 Fig — (A) Schematic representation of the predicted domain structure of DUSP. Domain boundaries and annotations were obtained from PFAM (via TriTrypDB release 68, May 2024). (B) Predicted three-dimensional structure of DUSP generated by AlphaFold (https://alphafold.ebi.ac.uk/entry/A4I9Z1). The protein is color-coded according to the per-residue confidence score (pLDDT), where blue indicates high confidence and orange indicates low confidence. (C) AlphaFold-predicted structure of DUSP colored by domain. (TIF) [file pntd.0014330.s004.tif]

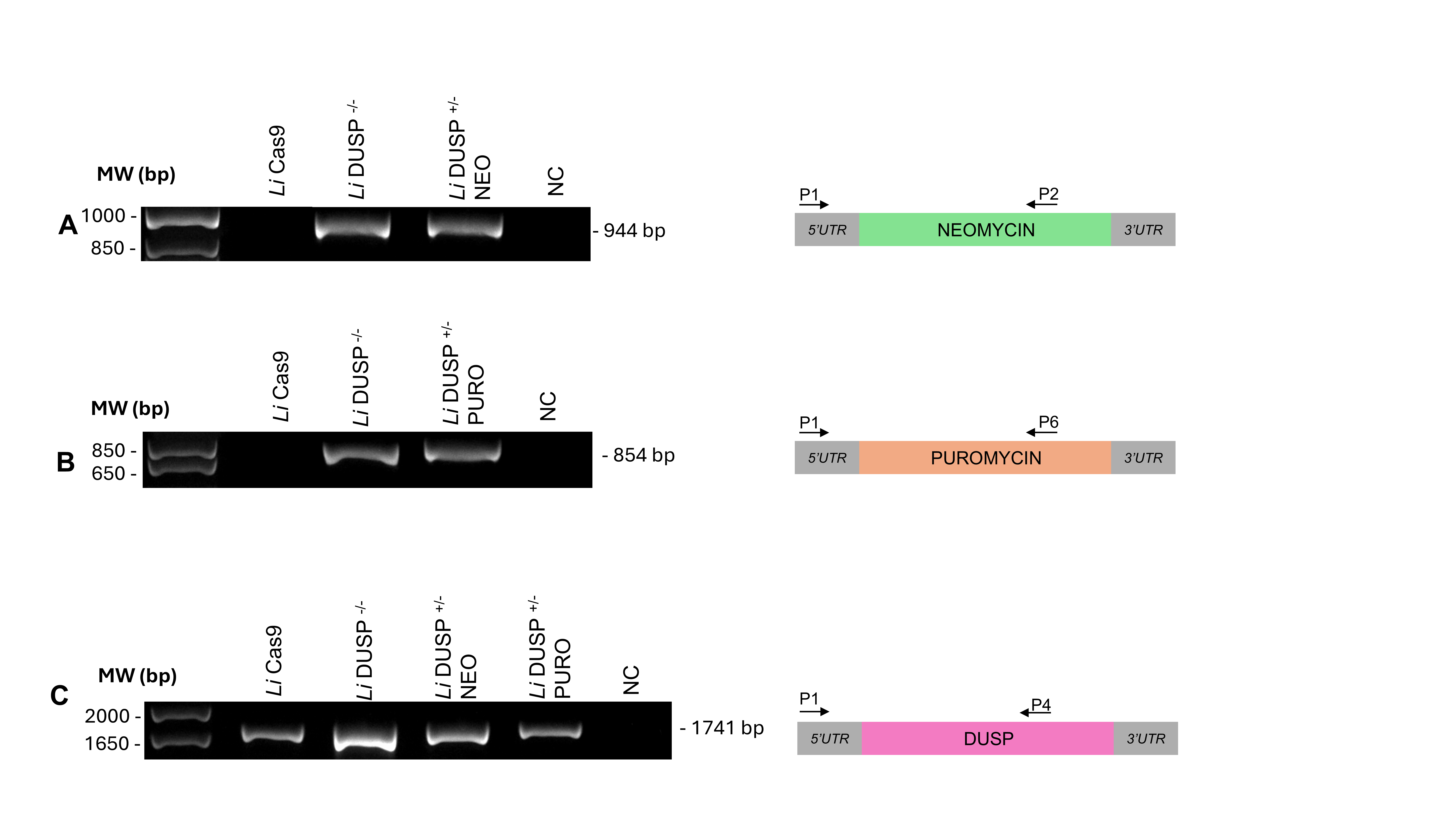

Supplement: S3 Fig — (A) The integration of the resistance marker neomycin (944 bp) and (B) puromycin (854 bp) was evaluated by PCR by annealing a primer in a 5′UTR region adjacent to the cassette (primer P1) and another primer annealed within resistance marker sequence (primer P2 and P6, respectively). (C) Fragment DUSP-coding sequence was amplified using PCR with a primer in a 5′UTR region adjacent to the cassette (primer P1) and primer P4. MW: molecular weight; NC: negative control; bp: base pair. (TIF) [file pntd.0014330.s005.tif]

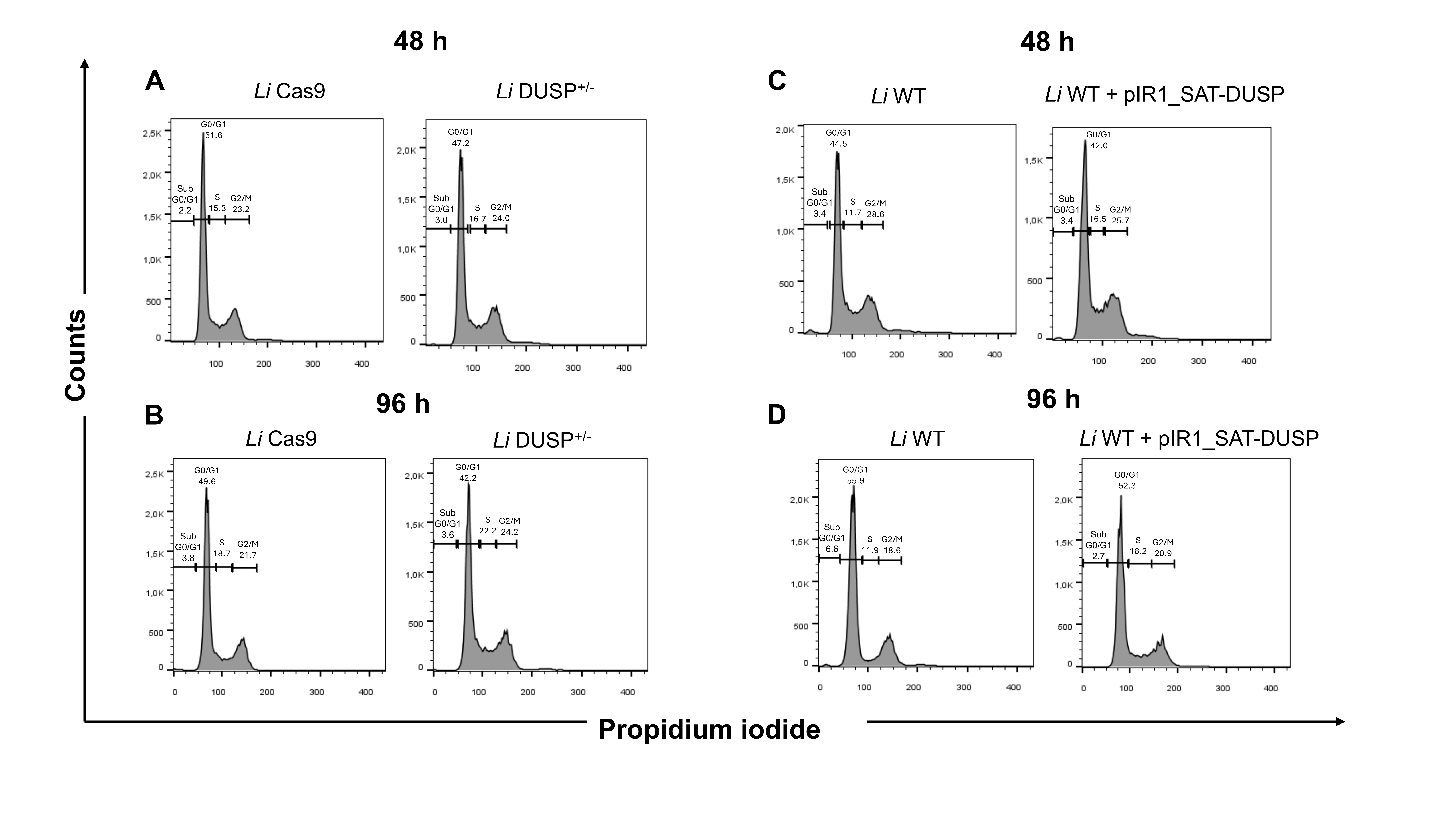

Supplement: S4 Fig — Cell cycle analysis of (A) and (B) DUSP heterozygous knockout at 48 hours and 96 hours, respectively. (C) and (D) cell cycle analysis of Li WT and Li WT + pIR1_SAT-DUSP parasites at 48 hours and 96 hours, respectively. The parasites were stained with propidium iodide and evaluation by flow cytometry. (TIF) [file pntd.0014330.s006.tif]
